# Mesoporous 2D covalent organic frameworks based on shape-persistent arylene-ethynylene macrocycles†
†Electronic supplementary information (ESI) available: Experimental procedures, TGA, FT-IR, PXRD and NMR spectra of compounds. See DOI: 10.1039/c5sc00894h


**DOI:** 10.1039/c5sc00894h

**Published:** 2015-05-06

**Authors:** Haishen Yang, Ya Du, Shun Wan, George Devon Trahan, Yinghua Jin, Wei Zhang

**Affiliations:** a Department of Chemistry and Biochemistry , University of Colorado Boulder , CO 80309 , USA . Email: wei.zhang@colorado.edu; b Storagenergy Technologies, Inc. , Salt Lake City , UT 84120 , USA

## Abstract

*Macrocycle-to-framework* strategy was explored to prepare covalent organic frameworks (COFs) using shape-persistent macrocycles as multitopic building blocks. We demonstrate well-ordered mesoporous 2D COFs (AEM–COF-**1** and AEM–COF-**2**) can be constructed from tritopic arylene-ethynylene macrocycles, which determine the topology and modulate the porosity of the materials. According to PXRD analysis and computer modelling study, these COFs adopt the fully eclipsed AA stacking mode with large accessible pore sizes of 34 or 39 Å, which are in good agreement with the values calculated by NLDFT modelling of gas adsorption isotherms. The pore size of COFs can be effectively expanded by using larger size of the macrocycles. Provided a plethora of polygonal shape-persistent macrocycles with various size, shape and internal cavity, *macrocycle-to-framework* strategy opens up a promising approach to expand the structural diversity of COFs and build hierarchical pore structures within the framework.

## Abstract 2

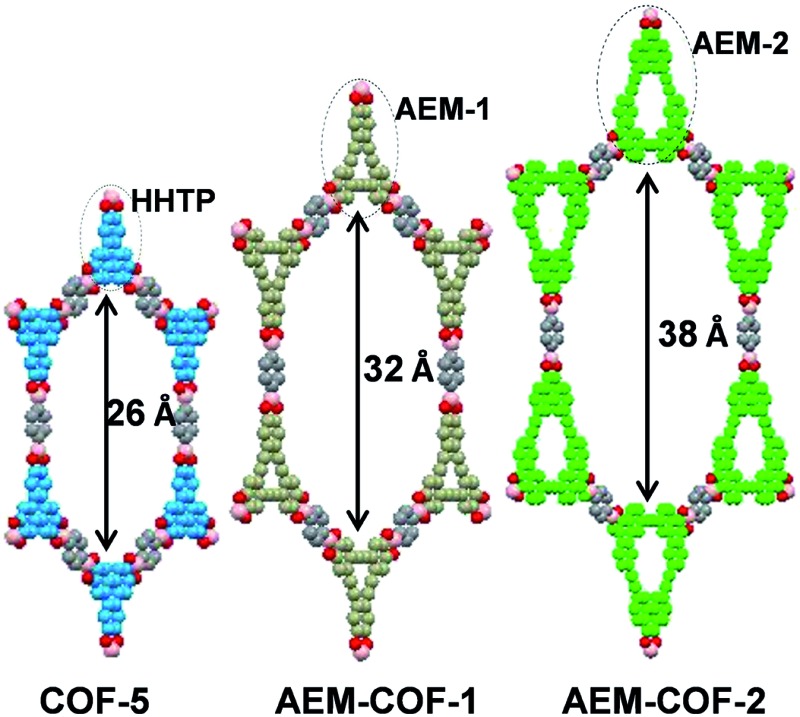
Covalent organic frameworks with high porosity and crystallinity have been synthesized, through macrocycle-to-framework strategy, using shape-persistent arylene-ethynylene macrocycles as the key components to control the topology and modulate the porosity.

## Introduction

Covalent organic frameworks (COFs) represent a novel class of porous crystalline polymers, in which the building blocks are assembled into two- or three-dimensional architectures through covalent bonds. COFs possess rigid structures, high thermal stabilities, and low densities. Since the pioneering work of Yaghi and co-workers,^1^ COFs have been intensively studied and applied in gas adsorption/separation,^2^–^6^ catalysis,^7^–^10^ and electronic devices.^11^–^14^ Similar to metal organic frameworks (MOFs), COFs are obtained through reticular synthesis, in which judiciously selected rigid building blocks are assembled to form ordered structures.^15^,^16^ The rigidity of building blocks is considered to be important to retain their geometrical features throughout the synthesis and form the predesigned solid-state frameworks. Although various COFs with different topologies and voids have been developed, so far, the building blocks are limited to multi-substituted aromatic molecules, such as benzene, pyrene, triphenylene, porphyrin, phthalocyanine, *etc.*^16^–^18^ Shape-persistent macrocycles (SPMs) represent an interesting group of rigid polygonal molecules with non-collapsible backbone structures. The structural rigidity of SPMs allows engineering of both their interior and exterior functionality, leading to their interesting applications, such as conducting molecular wires,^19^–^21^ sensors,^22^ light harvesting,^23^ and paramagnetic organic materials.^24^ We envision that SPMs can serve as a novel type of multitopic connectors for COFs. Potentially, COFs with hierarchical pore structures and diverse properties can be constructed by using SPMs with the pre-encoded intrinsic porosity and functionality. Although SPMs have unique advantages, they have rarely been explored in the preparation of COFs. Herein, as a proof-of-concept, we demonstrate the *macrocycle-to-framework* strategy for COF synthesis. Mesoporous 2D COFs with high surface area, large pore volume, good thermal stability and high crystallinity were successfully prepared from arylene-ethynylene macrocycles. By varying the size of macrocycles, the pore size of the COFs can be systematically tuned.

## Results and discussions

**Scheme 1:**
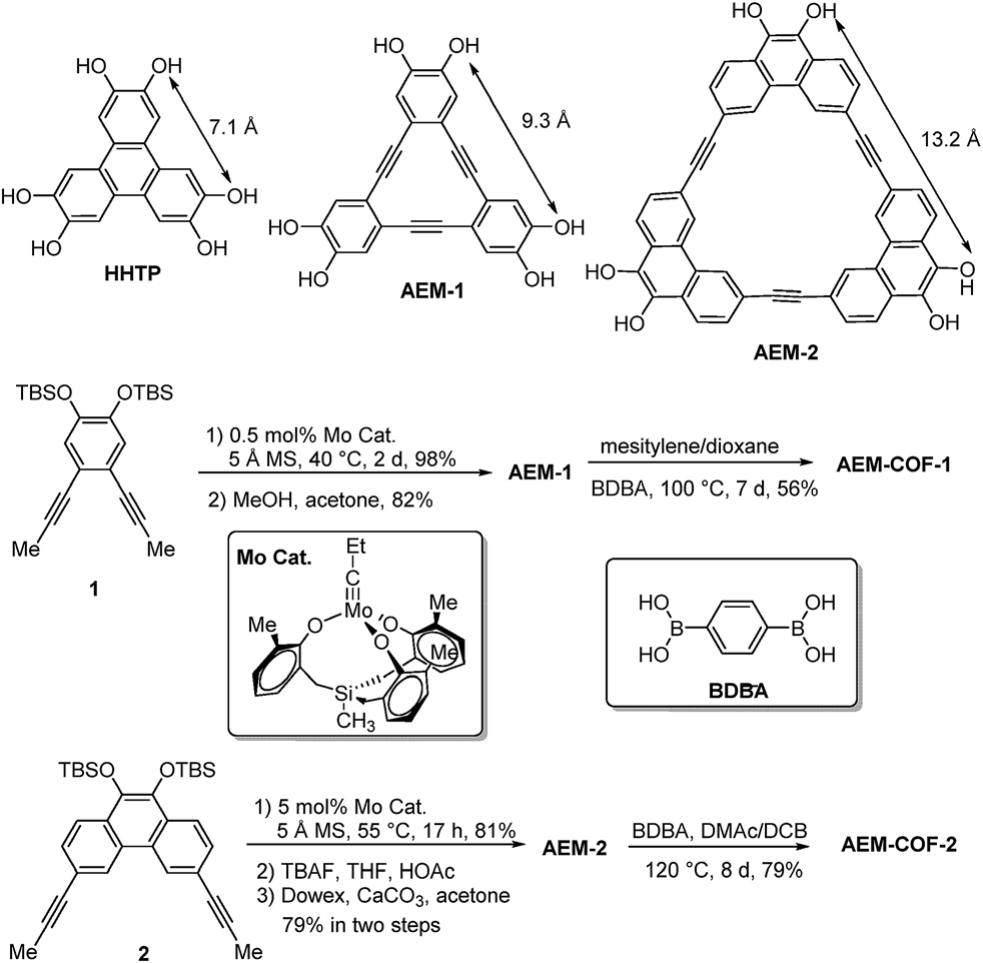
Synthesis of COFs containing AEMs.

Among various SPMs, arylene-ethynylene macrocycles (AEM) are of our particular interest, since they are perfectly planar and rigid and their size and geometry can be easily tailored.^25^,^26^ The well-known π–π interactions between macrocyclic building blocks would also favour the formation of COFs with high periodicity.^27^,^28^ Recent advent of dynamic covalent chemistry (DC_v_C),^25^,^29^,^30^ namely reversible alkyne metathesis, has opened an attractive, highly efficient strategy to build AEMs of various shapes and sizes. AEM-**1** and AEM-**2**, which closely resemble the commonly used trigonal connector, 2,3,6,7,10,11-hexahydroxytriphenylene (HHTP), were prepared (Scheme 1). AEM-**1** and AEM-**2** have similar triangle shape as HHTP (7 Å) but with increased sizes, having the extended lateral lengths of approximately 9 Å and 13 Å respectively. Gram-scale AEM-**1** and AEM-**2** were obtained from simple dipropynyl monomer **1**, or **2** through acyclic diyne metathesis macrocyclization (ADIMAC), followed by deprotection of TBS groups in high yields. Highly active multidentate triphenolsilane-based Mo(vi) carbyne complex^31^ was used as the catalyst for the metathesis reaction. Since alkyne metathesis is an equilibrium reaction, molecular sieves (5 Å) were added to scavenge 2-butyne byproduct and drive the equilibrium to the macrocycles. The dynamic covalent assembly approach employed herein has proven to be highly efficient compared to kinetically controlled irreversible cross-coupling approach (Sonogashira cross-coupling,^25^,^32^ Glaser coupling,^25^,^32^ Yamamoto cross-coupling^33^), which is generally associated with multi-step synthesis and low overall yields. Such one-step alkyne metathesis approach ensures easy accessibility of AEMs, making these macrocyclic building blocks practically useful for COF synthesis and further property study.

Most COFs are generally constructed from two types of building blocks: symmetric multitopic connectors and ditopic spacers. The multitopic connectors not only determine the topologies of the COFs, but also work in tandem with the spacers to determine the pore sizes, pore volumes, surface areas and functions of the COFs. Since there are a rich diversity of ditopic spacers readily available, a common strategy to enlarge pore apertures of COFs with a given topology has been to increase the length of the rigid ditopic linkers.^34^,^35^ The potential drawbacks of long ditopic linkers are the possibility of easy interpenetration and formation of fragile frameworks, which are frequently observed in MOFs.^36^–^38^ Here, we seek to develop an alternative approach, in which the dimensions of multitopic connectors are varied. We examined AEM-**1** and AEM-**2** as a novel type of multitopic building units, which can modulate pore size/distribution of COFs. We fixed the length of the linker using the same simple 1,4-benzenediboronic acid (BDBA), and varied the size of multitopic connectors: HHTP (7.1 Å), AEM-**1** (9.3 Å), and AEM-**2** (13.2 Å). For the comparison purpose, COF-5, which was previously reported by Yaghi,^1^ was also prepared following the literature procedure from BDBA and HHTP. We screened various solvent combinations and temperatures to find optimal conditions for the formation of ordered COFs. Crystalline AEM-COF-**1** was obtained in mesitylene/dioxane (1 : 1, v/v) by heating the reaction mixture at 100 °C for 7 days without stirring. Although AEM–COF-**2** shares a similar structure motif with AEM–COF-**1**, it requires a different solvent combination. A low surface area material was obtained when AEM-**2** and BDBA were heated (120 °C) in mesitylene/dioxane for 7 days. Among various solvent systems we tested (mesitylene/dioxane, DMF/mesitylene, DMAc/mesitylene, DMAc/DCB, *etc.*), the combination of DMAc/DCB provided crystalline AEM–COF-**2** with the highest surface area under conventional heating (7 days, 120 °C) or microwave heating (200 W, 120 °C, 40 min). AEM–COF-**1** and AEM–COF-**2** were isolated as yellow microcrystalline powders through centrifugation followed by successive washing with anhydrous acetone. Both COFs are insoluble in common organic solvents such as alkanes, arenes, acetone, ethers, and *N*,*N*-dimethylformamide.

**Fig. 1:**
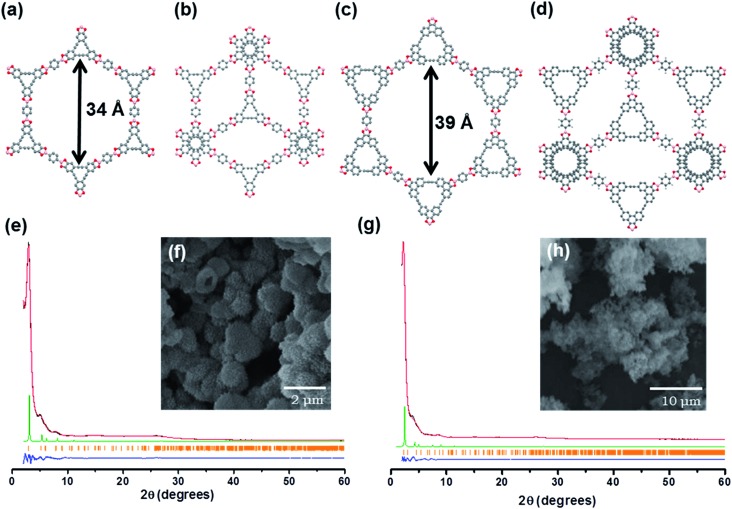
Structural representations of AEM–COF-**1**: bnn net (a), gra net (b); structural representations AEM–COF-**2**: bnn net (c), gra net (d); PXRD analysis of AEM–COF-**1** (e) and AEM–COF-**2** (g): observed pattern (black), the refined profile (red), the difference plot (blue), the observed reflections (orange), the calculated PXRD pattern from the proposed models (green); SEM images of AEM–COF-**1** (f) and AEM–COF-**2** (h).

AEM–COF-**1** and AEM–COF-**2** were characterized by FT-IR, ^13^C-MAS NMR, elemental analysis, TGA, SEM and PXRD analysis. The FT-IR spectra of AEM–COF-**1** and AEM–COF-**2** show stretching bands of B–O at 1335 cm^–1^ and 1323 cm^–1^, respectively. We also observed broad absorption band around 3430 cm^–1^, which likely corresponds to the residual hydroxyl groups of the macrocycles and boronic acids. In the magic angle spinning (MAS) solid-state ^13^C NMR spectrum of AEM–COF-**1**, we observed a single peak at 92.6 ppm which can be assigned to the carbons of triple bonds, indicating the uniformity of the chemical environment around C≡C bonds. The ^13^C NMR spectrum of AEM–COF-**2** shows C≡C bond carbon peak at 90.7 ppm. Thermogravimetric analysis (TGA) of AEM–COF-**1** and AEM–COF-**2** shows <10% weight loss at 400 °C and <30% at 800 °C under a nitrogen atmosphere (Fig. S2†), indicating the high thermal stability of these frameworks. The phase purities of AEM–COF-**1** and AEM–COF-**2** were confirmed to be single crystalline morphology by scanning electron microscopy (SEM) characterization (Fig. 1f and h).

The crystallinity of AEM–COF-**1** and AEM–COF-**2** was examined by powder X-ray diffraction (PXRD) measurement. The PXRD patterns of the COFs exhibit intense peak at 2*θ* = 2.9° and 2.2°, for AEM–COF-**1** and AEM–COF-**2**, respectively, along with some other peaks with lower diffraction intensities, indicating long-range molecular ordering in both COFs. We did not observe diffraction peaks that are characteristic for the starting materials (Fig. S5 and S6†). To elucidate the crystal lattice packing, a model was constructed using Materials Studio software package. Modelling was performed in the hexagonal system, with layers lying on the *ab* plane. Two extreme possibilities were evaluated, with respect to the stacking of the layers: (i) a fully eclipsed model with an AA stacking (space group *P*6/*mmm*), and (ii) a staggered model with an AB stacking (space group *P*6_3_/*mmc*). Each layer was translated from the next one by one-half of the *a* and *b* lattice parameters. A geometrical energy minimization was performed using the universal force-field implemented in the forcite module to optimize the geometry of the building molecules, as well as the unit cell parameters. The powder diffraction patterns for the models were then calculated and compared with the experimental ones. We found the simulated PXRD patterns of the fully eclipsed models of AEM–COF-**1** and AEM–COF-**2** are in excellent agreement with experimental results, indicating the eclipsed stacking mode of the layers (Fig. 1). A full profile pattern matching (Pawley) refinement in the Reflex module produced unit cell parameters for AEM–COF-**1**: *a* = *b* = 35.528 Å, *c* = 3.398 Å (residuals: *R*_p_ = 1.73% and *R*_wp_ = 2.33%); and AEM–COF-**2**: *a* = *b* = 40.935 Å, *c* = 3.257 Å (residuals: *R*_p_ = 2.06% and *R*_wp_ = 3.24%), both of which agree well with the observed reflections. Therefore, similar to COF-5, AEM–COF-**1** and AEM–COF-**2** adopt eclipsed stacking of the layers, which lead to 1D mesopores with theoretical diameters of 34 Å and 39 Å respectively.

**Fig. 2:**
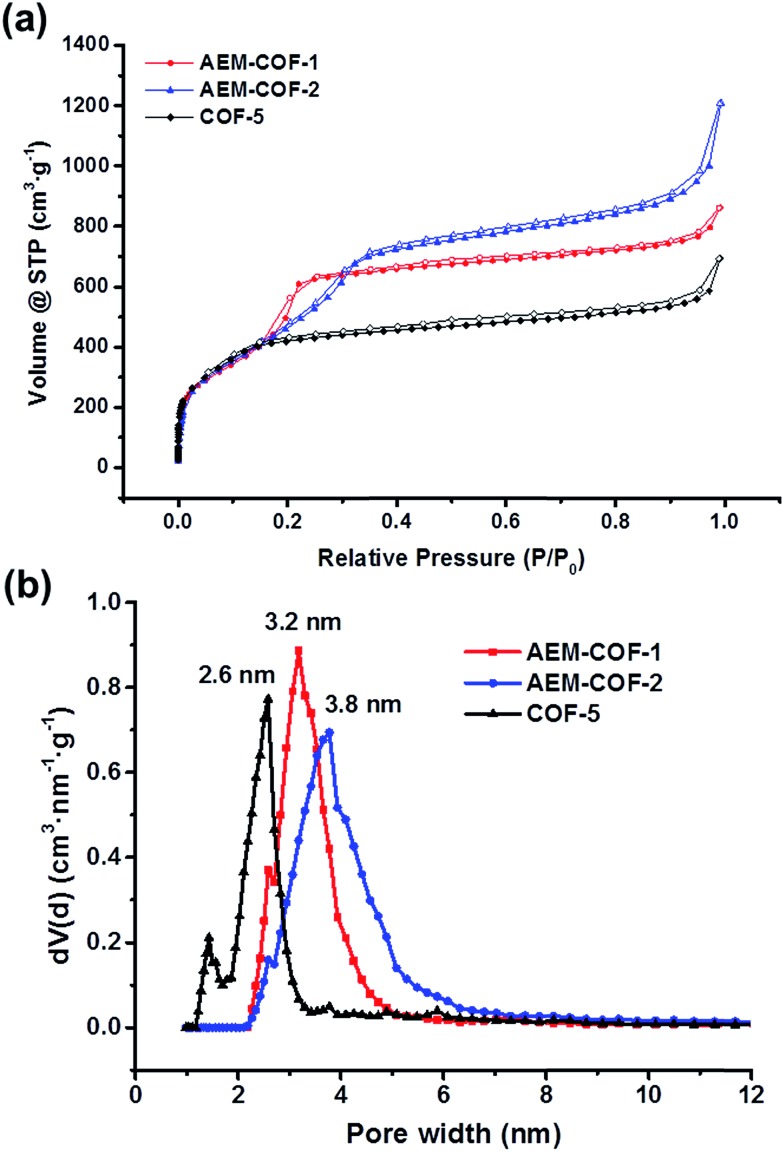
(a) Nitrogen gas adsorption isotherms measured at 77 K for AEM–COF-**1**, AEM–COF-**2**, and COF-5. Adsorption is labelled with filled symbols and desorption is labelled with hollow symbols. (b) Pore size distribution profiles for AEM–COF-**1**, AEM–COF-**2** and COF-5.

The porosities of the frameworks AEM–COF-**1** and AEM–COF-**2** were then investigated by N_2_ adsorption isotherms at 77 K and the results are summarized in Table 1. Prior to the porosity measurement, the samples were degassed at 100 °C under dynamic vacuum for 24 h. Under identical conditions, we also evaluated the gas adsorption properties of COF-5 prepared in our lab. All three frameworks exhibit reversible type IV nitrogen isotherms, which are typical for permanent mesoporous materials (Fig. 2a). We observed a sharp gas uptake at low pressure (*P*/*P*_0_ = 10^–5^ to 10^–2^) followed by a second stage pore filling starting around *P*/*P*_0_ = 0.05, which levels off at a relative pressure of *P*/*P*_0_ = 0.18, 0.25 and 0.35 for COP-5, AEM–COF-**1** and AEM–COF-**2**, respectively. The gradual shift of the step positions suggests the increasing sizes of the pores in these three COFs. Calculations based on the non-local density functional theory (NLDFT) also reveal the trend of increasing pore sizes in the series, showing a narrow pore-size distribution (PSD) centered around 2.6 nm for COF-5, 3.2 nm for AEM–COF-**1**, and 3.8 nm for AEM–COF-**2** (Fig. 2b). These values are consistent with the theoretical pore sizes (2.7 nm, 3.4 nm, and 3.9 nm for COF-5, AEM–COF-**1**, and AEM–COF-**2**, respectively) predicted from the modelling based on XRD crystal packing. Correspondingly, we observed increasing pore volumes (*V*_p_), which were calculated to be 0.828 cm^3^ g^–1^ (COF-5), 1.15 cm^3^ g^–1^ (AEM–COF-**1**), and 1.38 cm^3^ g^–1^ (AEM–COF-**2**) at *P*/*P*_0_ = 0.90. No or little hysteresis loops were observed in the whole range of adsorption–desorption isotherms in all three frameworks. The absence of hysteresis loop has been observed for similar mesoporous MCM-41 with tubular hexagonal pores of sizes <40 Å at temperatures above 77.4 K.^39^,^40^ The thermodynamic theory predicts that the size of the hysteresis loop decreases with increasing the temperature or decreasing pore diameters,^41^ supporting the presence of small mesopores of sizes 25–40 Å in our frameworks. The BET surface area of COF-5 was calculated to be 1517 m^2^ g^–1^ (correlation coefficient = 0.998), which is in good agreement with the reported literature value (1590 m^2^ g^–1^).^1^ Similar calculated BET surface areas were observed for AEM–COF-**1** (1445 m^2^ g^–1^, correlation coefficient = 0.998) and AEM–COF-**2** (1489 m^2^ g^–1^, correlation coefficient = 0.999). As shown in Table 1, our study clearly demonstrates the feasibility of *macrocycle-to-framework* strategy to construct ordered crystalline COFs with tunable pore diameters and volumes by varying the dimensions of tritopic macrocyclic building units.

**Table 1:** Comparison of the porosity of COFs

COFs	SA_BET_^*a*^	*V*_Total_ ^*b*^	Pore size	
			Predicted^*c*^	Experimental^*d*^
COF-5	1517	0.83	2.7	2.6
AEM–COF-**1**	1445	1.15	3.4	3.2
AEM–COF-**2**	1487	1.38	3.9	3.8

^*a*^Surface area (m^2^ g^–1^) calculated from the nitrogen adsorption based on the BET model.

^*b*^The total pore volume (cm^3^ g^–1^) calculated at *P*/*P*_0_ = 0.90.

^*c*^Predicted pore size based on the eclipsed stacking of layers.

^*d*^Calculated pore size from nitrogen adsorption isotherms using NLDFT-N_2_-silica adsorption branch kernel at 77 K based on a cylindrical pore model.

Initially, we expected hierarchical pore structures in the case of AEM–COF-**2**, which contains AEM-**2** with interior void of 5.8 Å. However, we did not observe micropores below 1 nm range. Although the X-ray diffraction data is in excellent agreement with the perfectly eclipsed model of the AEM–COF-**2** layered structure, there might be slight offset between the adjacent interlayers, leading to the restricted accessibility of such micropores. In order to obtain COFs with multiple-type pore structures, the use of macrocycles with large intrinsic pores are desired.

## Conclusions

We have demonstrated that arylene-ethynylene macrocycles (AEMs) can be utilized as well-defined building blocks for construction of COFs with high thermal stability, permanent porosity, and high crystallinity, either under conventional solvothermal conditions or microwave heating. The π–π interactions between rigid arylene-ethynylene backbones likely contribute considerably to the eclipsed packing of the layers as well as the formation of ordered crystalline materials. Our study shows that the customizable SPMs can be effectively utilized as a new type of multitopic connectors to control the topologies of the COFs and tune the surface area, pore size, and pore volume of the COFs. Given the vast availability of SPMs with different backbones and properties, *e.g.* arylene-vinylene macrocycles (AVM),^42^ arylene-ethynylene macrocycles (AEM),^25^,^32^,^43^ and aryl amide macrocycles (AAM),^44^,^45^ such *macrocycle-to-framework* strategy opens up new avenues in the synthesis of COFs with intriguing architectures, properties and applications.

## Supplementary Material

Supplementary information

## References

[cit1] Côté A. P., Benin A. I., Ockwig N. W., O'Keeffe M., Matzger A. J., Yaghi O. M. (2005). Science.

[cit2] Mendoza-Cortes J. L., Goddard W. A., Furukawa H., Yaghi O. M. (2012). J. Phys. Chem. Lett..

[cit3] Mendoza-Cortés J. L., Han S. S., Furukawa H., Yaghi O. M., Goddard W. A. (2010). J. Phys. Chem. A.

[cit4] Doonan C. J., Tranchemontagne D. J., Glover T. G., Hunt J. R., Yaghi O. M. (2010). Nat. Chem..

[cit5] Furukawa H., Yaghi O. M. (2009). J. Am. Chem. Soc..

[cit6] Han S. S., Furukawa H., Yaghi O. M., Goddard W. A. (2008). J. Am. Chem. Soc..

[cit7] Pachfule P., Panda M. K., Kandambeth S., Shivaprasad S. M., Diaz D. D., Banerjee R. (2014). J. Mater. Chem. A.

[cit8] Fang Q. R., Gu S., Zheng J., Zhuang Z. B., Qiu S. L., Yan Y. S. (2014). Angew. Chem., Int. Ed..

[cit9] Ding S.-Y., Gao J., Wang Q., Zhang Y., Song W.-G., Su C.-Y., Wang W. (2011). J. Am. Chem. Soc..

[cit10] Chen X., Huang N., Gao J., Xu H., Xu F., Jiang D. (2014). Chem. Commun..

[cit11] Jin S., Ding X., Feng X., Supur M., Furukawa K., Takahashi S., Addicoat M., El-Khouly M. E., Nakamura T., Irle S., Fukuzumi S., Nagai A., Jiang D. (2013). Angew. Chem., Int. Ed..

[cit12] Wan S., Guo J., Kim J., Ihee H., Jiang D. L. (2008). Angew. Chem., Int. Ed..

[cit13] Wan S., Guo J., Kim J., Ihee H., Jiang D. L. (2009). Angew. Chem., Int. Ed..

[cit14] DeBlase C. R., Silberstein K. E., Truong T.-T., Abruña H. D., Dichtel W. R. (2013). J. Am. Chem. Soc..

[cit15] Ding S. Y., Wang W. (2013). Chem. Soc. Rev..

[cit16] Feng X., Ding X., Jiang D. (2012). Chem. Soc. Rev..

[cit17] Zou X., Ren H., Zhu G. (2013). Chem. Commun..

[cit18] Colson J. W., Dichtel W. R. (2013). Nat. Chem..

[cit19] Luo J., Yan Q., Zhou Y., Li T., Zhu N., Bai C., Cao Y., Wang J., Pei J., Zhao D. (2010). Chem. Commun..

[cit20] Nakao K., Nishimura M., Tamachi T., Kuwatani Y., Miyasaka H., Nishinaga T., Iyoda M. (2006). J. Am. Chem. Soc..

[cit21] Błaszczyk A., Chadim M., von Hänisch C., Mayor M. (2006). Eur. J. Org. Chem..

[cit22] Naddo T., Che Y., Zhang W., Balakrishnan K., Yang X., Yen M., Zhao J., Moore J. S., Zang L. (2007). J. Am. Chem. Soc..

[cit23] Höger S. (2010). Pure Appl. Chem..

[cit24] Hui P., Chandrasekar R. (2013). Adv. Mater..

[cit25] Zhang W., Moore J. S. (2006). Angew. Chem., Int. Ed..

[cit26] Höger S. (2004). Chem.–Eur. J..

[cit27] Zhang J., Moore J. S. (1992). J. Am. Chem. Soc..

[cit28] Zhang J., Moore J. S. (1994). J. Am. Chem. Soc..

[cit29] Corbett P. T., Leclaire J., Vial L., West K. R., Wietor J. L., Sanders J. K. M., Otto S. (2006). Chem. Rev..

[cit30] Jin Y., Yu C., Denman R. J., Zhang W. (2013). Chem. Soc. Rev..

[cit31] Yang H., Liu Z., Zhang W. (2013). Adv. Synth. Catal..

[cit32] Zhao D., Moore J. S. (2003). Chem. Commun..

[cit33] Schlütter F., Rossel F., Kivala M., Enkelmann V., Gisselbrecht J.-P., Ruffieux P., Fasel R., Müllen K. (2013). J. Am. Chem. Soc..

[cit34] Feng X., Dong Y. P., Jiang D. L. (2013). CrystEngComm.

[cit35] Spitler E. L., Colson J. W., Uribe-Romo F. J., Woll A. R., Giovino M. R., Saldivar A., Dichtel W. R. (2012). Angew. Chem., Int. Ed..

[cit36] Park Y. K., Choi S. B., Kim H., Kim K., Won B.-H., Choi K., Choi J.-S., Ahn W.-S., Won N., Kim S., Jung D. H., Choi S.-H., Kim G.-H., Cha S.-S., Jhon Y. H., Yang J. K., Kim J. (2007). Angew. Chem., Int. Ed..

[cit37] Eddaoudi M., Kim J., Rosi N., Vodak D., Wachter J., O'Keeffe M., Yaghi O. M. (2002). Science.

[cit38] Lin X., Telepeni I., Blake A. J., Dailly A., Brown C. M., Simmons J. M., Zoppi M., Walker G. S., Thomas K. M., Mays T. J., Hubberstey P., Champness N. R., Schröder M. (2009). J. Am. Chem. Soc..

[cit39] Ravikovitch P. I., ODomhnaill S. C., Neimark A. V., Schuth F., Unger K. K. (1995). Langmuir.

[cit40] Neimark A. V., Ravikovitch P. I., Domhnaill S. C. Ó., Schüth F. and Unger K. K., in Fundamentals of Adsorption, ed. M. D. LeVan, Springer US, 1996, vol. 356, ch. 83, pp. 667–674.

[cit41] Evans R., Marconi U. M. B., Tarazona P. (1986). J. Chem. Soc., Faraday Trans..

[cit42] Jin Y., Zhang A., Huang Y., Zhang W. (2010). Chem. Commun..

[cit43] Gross D. E., Zang L., Moore J. S. (2012). Pure Appl. Chem..

[cit44] Fu H., Liu Y., Zeng H. (2013). Chem. Commun..

[cit45] Kline M., Wei X., Gong B. (2013). Org. Lett..

