# Supplementary material for: Mesoporous 2D covalent organic frameworks based on shape-persistent arylene-ethynylene macrocycles
Source: Chem Sci. 2015 May 6;6(7):4049–53. doi: 10.1039/c5sc00894h (PMC5707634; doi:10.1039/c5sc00894h)
Supplement: Supplementary file 1 [file SC-006-C5SC00894H-s001.pdf]

Electronic Supplementary Material (ESI) for Chemical Science.
This journal is © The Royal Society of Chemistry 2015

Supporting Information for

Mesoporous 2D Covalent Organic Frameworks Based on Shape-Persistent Arylene-Ethynylene Macrocycles

Haishen Yang,[a] Ya Du,[a] Shun Wan,[b] George Devon Trahan,[a] Yinghua Jin,[a] and Wei Zhang*[a]

[a] Department of Chemistry and Biochemistry, University of Colorado Boulder, CO 80309, USA.

[b] Storagenergy Technologies, Inc. Salt Lake City, UT 84120, USA.

Tel: (+1) 303-492-0652; Fax: (+1) 303-492-5894; E-mail: wei.zhang@colorado.edu

Table of Contents

1. Materials and Methods S2
2. Synthetic procedures of AEM-1 and AEM-2 .... S3
3. Synthetic procedures for preparation of AEM-COF-1 and AEM-COF-2 .... S6
4. Thermal gravimetric analysis of COF-5, AEM-COF-1 and AEM-COF-2 .... S7
5. FT-IR spectra of AEM-COF-1, AEM-1 and BDBA S8
6. Peak assignments for FT-IR spectrum of AEM-COF-1 .... S8
7. FT-IR spectra of AEM-COF-2, AEM-2 and BDBA S9
8. Peak assignments for FT-IR spectrum of AEM-COF-2 .... S10
9. Powder X-Ray Diffraction Patterns of AEM-COF-1, AEM-1, and BDBA S11
10. Powder X-Ray Diffraction Patterns of AEM-COF-2, AEM-2, and BDBA S12
11. Structural Modeling and X-ray Diffraction Analyses. S12
12. Solid State NMR spectra of the as synthesized AEM-COF-1 and AEM-COF-2 .... S14
13. ^{1}H and ^{13}C NMR spectra of the new compounds S15

1. Materials and Methods

Unless otherwise stated, all commercially available chemical reagents were used without further purification. All reactions were conducted under inert atmosphere (nitrogen or argon).

Flash column chromatography was performed by using a 100-150 times weight excess of flash silica gel 32-63 μm from Dynamic Absorbants Inc. Fractions were analyzed by TLC using TLC silica gel F_{254} 250 μm precoated-plates from SiliCycle Inc.

NMR spectra were taken on Inova 400 and Inova 500 spectrometers. Solid-state cross polarization magic angle spinning (CP/MAS) NMR spectra were recorded on an Inova 400 NMR spectrometer. Deuterated solvents were purchased from Cambridge Isotope Laboratories (Andover, MA) and used as received. MALDI-TOF spectra were recorded on a Voyager-DE™ STR Biospectrometry Workstation using Sodium trifluoroacetate as the matrix.

The FT-IR spectra were obtained in the form of KBr pellets, using a Thermo Nicolet Avatar-370 spectrometer. Elemental analyses were taken at Huffman Laboratories, Inc. Thermogravimetric analysis (TGA) was performed in Pyris 7 TGA (Perkin Elmer) by heating the sample under an atmosphere of nitrogen from 50 ˚C to 800 ˚C at 10 ˚C/min.

The Quantachrome Autosorb ASiQ automated gas sorption analyzer was used to measure N_2, adsorption isotherms. The samples were activated by heating at 100 ˚C under the vacuum for at least 22 hours. Ultra high purity grade (99.999%) N_2, oil-free valves and gas regulators were used for all free space corrections and measurements. For all of the gas adsorption measurements, the temperatures were controlled by using a refrigerated bath of liquid N_2 (77 K).

Scanning Electron Microscopy images (SEM) were recorded using a JSM-6480LV (LVSEM) at 5.0 kV.

2. Synthetic procedures of AEM-1 and AEM-2

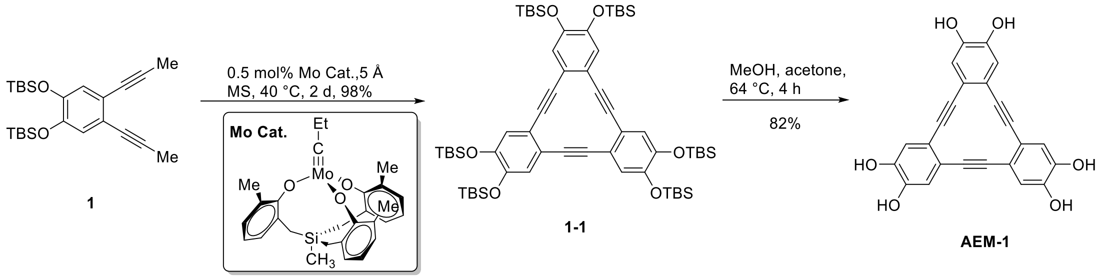

Synthesis of compound 1-1: Compound **1-1** was prepared in multi-gram scale following our previously reported procedure with decreased catalyst loading of 0.5 mol%. The ligand (25 mg, 0.061 mmol) and the precursor (40 mg, 0.061 mmol) were premixed in dry carbon tetrachloride (10 mL) and stirred for 15 minutes to generate the catalyst *in situ*. A solution of the compound **1** (5.07 g, 12.2 mmol) in CCl_4 (60 mL), and 5 Å molecular sieves (15 g), were added to the above catalyst solution and the mixture was stirred at 40 ˚C for 2 d. The molecular sieves were filtered and washed thoroughly with CH_2Cl_2. The filtrate was concentrated, and the residue was purified by flash column chromatography using hexane/ethyl acetate (v:v, 50:1) as the eluent to give the product **1-1** (4.31 g, 98%): ^{1}H NMR (500 MHz, $CDCl_3$) δ 6.73 (s, 6H), 0.99 (s, 54H), 0.22 (s, 36H). The ^{1}H NMR data is consistent with the literature report.[1]

Synthesis of compound AEM-1: A mixture of **1-1** (1.69 g, 1.56 mmol), HCl (conc. 37%, 1.0 mL), acetone (8 mL), and MeOH (12 mL) was stirred at 55 ˚C for 4 h. The solution was concentrated under reduced pressure. Then the residue was washed successively with water, hexanes, and water to give the product (0.510 g, 82%): ^{1}H NMR (500 MHz, acetone-d_6) δ 8.47 (d, J = 4.4 Hz, 6H), 6.73 (s, 6H); ^{13}C NMR (75 MHz, acetone-d$_6$) δ 206.1, 146.6, 119.8, 119.0, 91.9; HRMS (ESI): calcd. for $C_{24}H_{12}O_6Li^+$ [M+Li$^+$]: 403.0788. Found: 403.0796.

In order to compare the packing modes of AEM-1 with the resulting polymer AEM-COF-1, we explored the recrystallization of AEM-1 under various conditions. Single crystals of AEM-1 suitable for X-ray crystallography analysis were obtained by vapor diffusion method at room temperature in dioxane/hexanes. The packing structure shows that there are four crystallographically unique macrocycles, which arrange themselves to form layers. However, no direct π-stacking or H-bonding interactions between two neighboring macrocycles were observed. The crystal structure contains around

20 dioxane solvent molecules per two macrocycles. Presumably, the interactions between dioxane oxygen and hydroxyl group of the AEM-1 are the primary driving force in the case of AEM-1 crystallization.

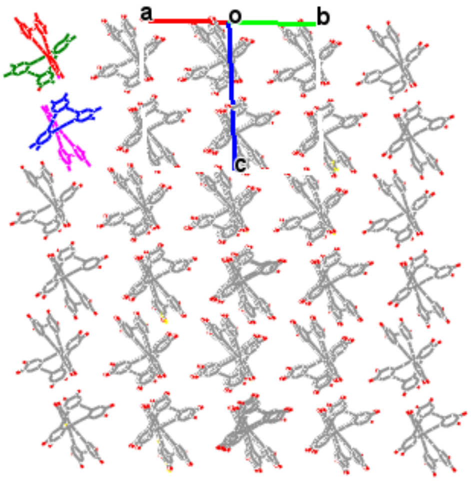

Figure S1. Crystal packing view of AEM-1 along (110) plane. Dioxane solvent molecules are omitted for clarity.

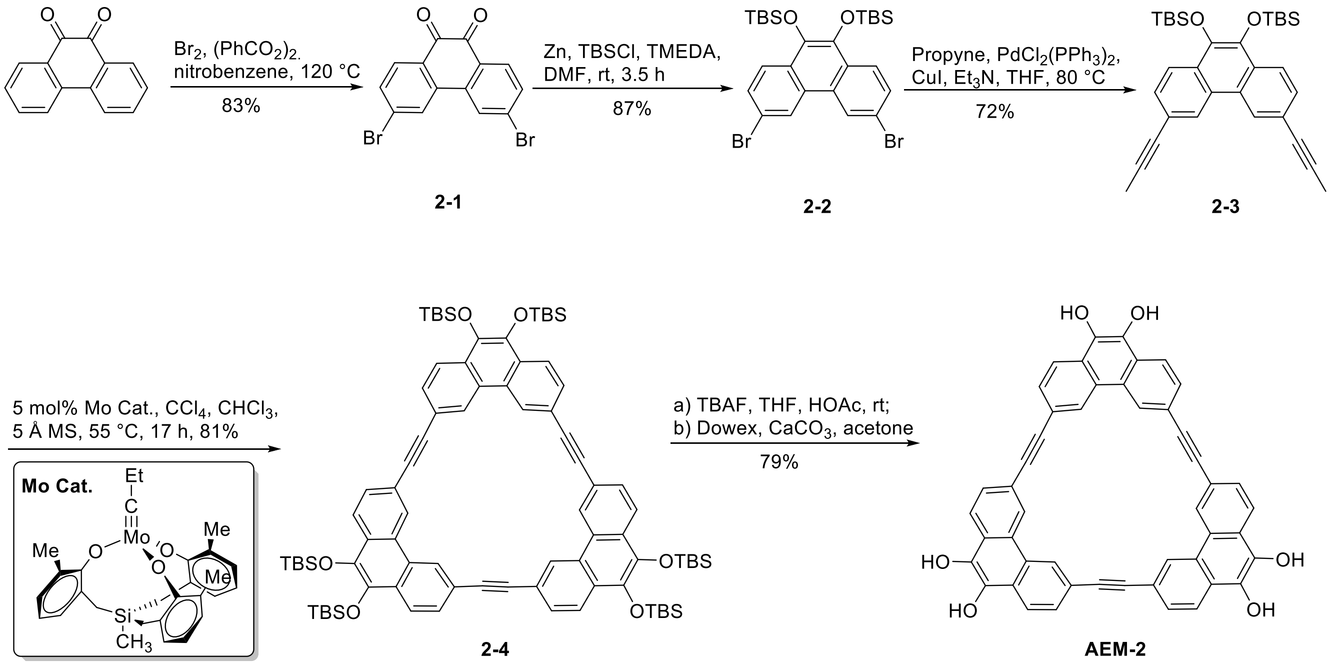

Synthesis of compound 2-1: Compound **2-1** was synthesized from phenanthrene-9,10-dione according to the reported procedure.[2] ^{1}H NMR (400 MHz, $CDCl_3$) δ 8.12 (d, J = 1.7 Hz, 2H), 8.08 (d, J = 8.3 Hz, 2H), 7.67 (dd, J = 8.3, 1.7 Hz, 2H). The ^{1}H NMR data is consistent with the literature report.[2]

Synthesis of compound 2-2: Compound **2-2** was synthesized according to reported procedure.[3] ^{1}H NMR (500 MHz, $CDCl_3$) δ 8.61 (d, J = 1.9 Hz, 2H), 8.05 (d, J = 8.8 Hz, 2H), 7.67 (dd, J = 8.8, 1.9 Hz, 2H), 1.12 (s, 18H), 0.06 (s, 12H). The ^{1}H NMR data is consistent with the literature report.[3]

Synthesis of compound 2-3: Propyne was bubbled through a degassed mixture of dibromide **2-2** (3.25 g, 5.45 mmol), $PdCl_2(PPh_3)_2$ (191mg, 0.27 mmol), CuI (21 mg, 0.11 mmol), Et_3N (7.6 mL, 54.5 mmol) and THF (55 mL) in a schlenk tube for ca. 5 min. Then the tube was sealed and heated for 22 h at 80 ˚C. CH_2Cl_2 (100 mL) and water (100 mL) were added. The organic phase was separated and the aqueous phase was extracted with CH_2Cl_2 (2 x 100 mL). The combined organic extracts were washed with water (100 mL) and brine (100 mL) and dried over anhydrous Na_2SO_4. The solvent was removed under reduced pressure. Purification of the residue by flash chromatography (hexanes/CH_2Cl_2, 8:1) yielded the title compound as a yellow solid (2.01 g, 72%): ^{1}H NMR (500 MHz, $CDCl_3$) δ 8.61 (d, J = 1.5 Hz, 2H), 8.06 (d, J = 8.5 Hz, 2H), 7.54 (dd, J = 8.5, 1.5 Hz, 2H), 2.13 (s, 6H), 1.12 (s, 18H), 0.06 (s, 12H); ^{13}C NMR (75 MHz, $CDCl_3$) δ 137.9, 129.6, 129.0, 126.9, 125.8, 122.9, 120.5, 86.0, 80.3, 26.5, 18.7, 4.5, -3.5. HRMS (ESI): calcd. for $C_{32}H_{42}O_2^+$ [M^+]: 514.2718. Found: 514.2716.

Synthesis of compound 2-4: The ligand (**$L_{2,Me}$**) (36 mg, 0.087 mmol) and the precursor (58 mg, 0.087 mmol) were premixed in dry carbon tetrachloride (10 mL) and stirred for 15 minutes to generate the catalyst (**Mo. Cat.**) *in situ*. Subsequently, compound **2-3** (0.90 g, 1.75 mmol), 5 Å molecular sieves (5.25 g) and $CHCl_3$ (20 mL) were added, and stirring was continued at 55 °C for 17 h. The 5 Å molecular sieves were removed by filtration and washed with CH_2Cl_2 (~ 30 mL). The filtrate was concentrated under reduced pressure. Purification of the residue by flash column chromatography (hexanes/CH_2Cl_2 = 10:1) yielded the compound **2-4** (0.657 g, 81%) as a waxy solid: ^{1}H NMR (500 MHz, $CDCl_3$) δ 8.61 (d, J = 1.5 Hz, 2H), 8.06 (d, J = 8.5 Hz, 2H), 7.54 (dd, J = 8.5, 1.5 Hz, 2H), 2.13 (s, 6H), 1.12 (s, 18H), 0.06 (s, 12H); ^{13}C NMR (75 MHz, $CDCl_3$) δ 138.5, 130.1, 128.7, 127.2, 126.8, 123.2, 120.0, 90.9, 26.5, 18.8, -3.4; HRMS (ESI): calcd. for $C_{84}H_{108}O_6Si_6Li^+$ [$M+Li^+$]: 1387.6916. Found: 1387.6943.

Synthesis of compound AEM-2: A solution of TBAF (1 M, 9.5 mL, 9.5 mmol) was added to a Schlenk tube. It was degassed under vacuum, and air-free THF (40 mL) and HOAc (0.6 mL, 10 mmol) were added. Then a solution of **2-4** (1.44 g, 1.04 mmol) in THF (60 mL) was added dropwise at rt. The mixture was stirred at rt for 23 h. The solid was filtered and washed with THF (3 × 12 mL). The solid was redissolved in acetone (110 mL), and DOWEX 50WX8-400 (6 g) and $CaCO_3$ (2.4 g) were added. The mixture was stirred at rt for 17 h, and filtered through a frit funnel. The filtrate was concentrated under reduced pressure to give the product (0.57 g, 1.04 mmol): ^1H NMR (500 MHz, DMSO-d_6) δ 9.44 (s, 6H), 9.30 (d, J = 2.0 Hz, 6H), 8.23 (d, J = 8.4 Hz, 6H), 7.81 (dd, J = 8.6, 1.4 Hz, 6H); ^{13}C NMR (75 MHz, DMSO-d_6) δ 135.9, 128.8, 127.4, 126.3, 125.4, 122.2, 118.4, 90.6; MALDI-TOF (m/z): $[M+H]^+$ calcd. for $C_{48}H_{25}O_6^+$, 697.16. found: 697.70.

3. Synthetic procedures for preparation of AEM-COF-1 and AEM-COF-2

Synthesis of AEM-COF-1: A mixture of AEM-**1** (42 mg, 0.11 mmol) and BDBA (24 mg, 0.15 mmol) in mesitylene/dioxane (8 mL, 1/1 by vol.) was degassed in a 25-mL Schlenk tube by three freeze-pump-thaw cycles. The tube was sealed and heated at 100 ºC for 7 days. The precipitate was collected by centrifugation, washed with anhydrous acetone, and dried at 120 ºC under vacuum overnight. The final product was obtained as a yellow solid (32 mg, 56%): Elemental Analysis for $(C_{11}H_4BO_2)_n$: C (71.33), H (3.48), B (4.42); Cacl.: C (73.83), H (2.25), B (6.04).

Synthesis of AEM-COF-2

Conventional heating: A mixture of AEM-**2** (35 mg, 0.05 mmol) and BDBA (12.4 mg, 0.075 mmol) in DMAc/DCB (4.4 mL, 1/1 by vol.) was degassed in a 25-mL Schlenk tube by three freeze-pump-thaw cycles. The tube was sealed and heated at 120 ºC for 8 days. The precipitate was collected by centrifugation, washed with anhydrous acetone, and dried under vacuum overnight, to give a yellow solid (33 mg, 79%). Elemental Analysis for $(C_{19}H_8BO_6)_n$: C (75.65), H (3.41), B (2.14); Cacl.: C (81.77), H (2.89), B (3.87).

Microwave heating: AEM-**2** (35 mg, 0.05 mmol) and BDBA (12.4 mg, 0.075 mmol) were added into a 10 mL microwave tube, followed by degassed DMAc/DCB (4.4 mL, 1/1 by vol.). The mixture was heated under microwave irradiation (200 W, 120 ºC, 40 min). The precipitate was collected by centrifugation, washed with anhydrous acetone, and dried under vacuum overnight, to give a yellow solid (34 mg, 81%).

4. Thermal gravimetric analysis of COF-5, AEM-COF-1 and AEM-COF-2

Samples were run on a TA Instruments Q-500 series thermal gravimetric analyzer with samples held in a platinum pan under nitrogen atmosphere. A 10 K min^{-1} ramp rate was used.

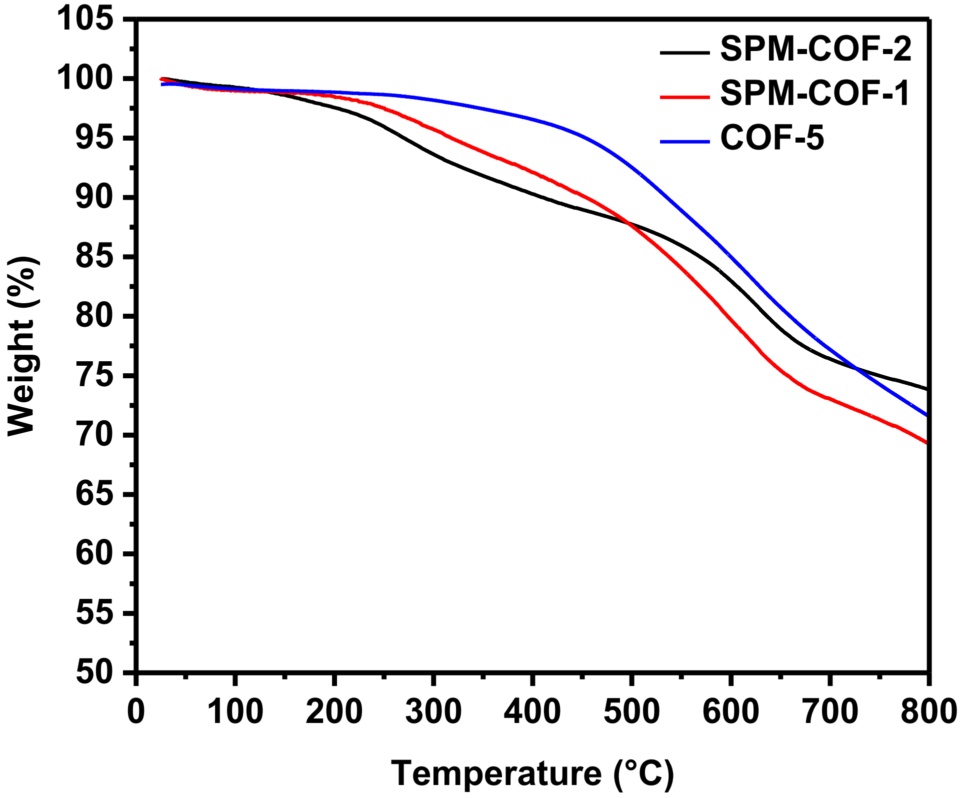

Figure S2. TGA of COF-5, AEM-COF-**1** and AEM-COF-**2.**

5. FT-IR spectra of AEM-COF-1, AEM-1 and BDBA

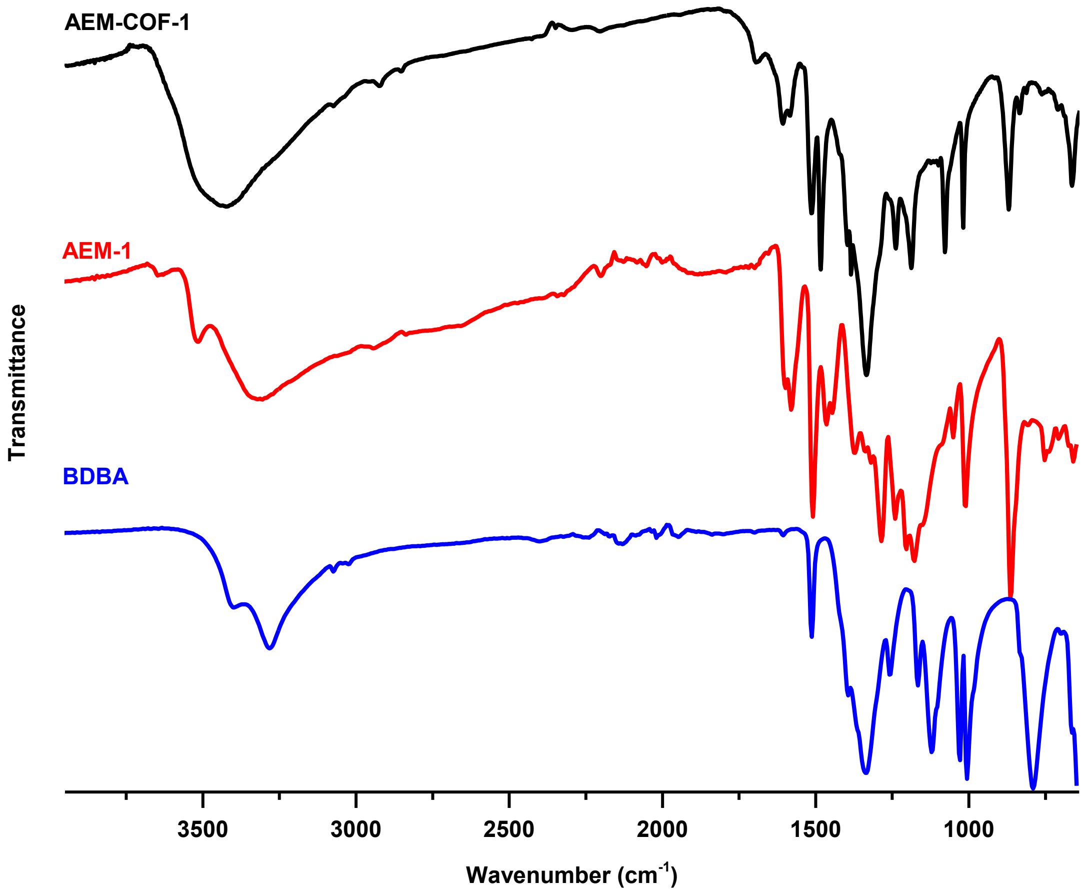

Figure S3. FT-IR spectra of "as synthesized" AEM-COF-**1**, AEM-**1** and BDBA.

6. Peak assignments for FT-IR spectrum of AEM-COF-1

Table S1. Peak assignments for FT-IR spectrum of AEM-COF-**1**

Peak (cm^{-1})	Assignment and Notes
3423.2 (m)	O-H stretch from or end $-B(OH)_2$ groups at the surface of crystallites.
3073.1 (w)	Aromatic C−H stretch from benzene 1,4-diboronic acid phenyl group of AEM-COF-**1**; *cf.* band from benzene 1,4-diboronic acid at 3074.3 (w).
2954.6 (w)	C−H stretching from included guest molecules and triphenylene building block.
2923.1 (w)	
2852.8 (w)	
1606.5 (w)	C=C stretch in typical region for fused aromatics. Also present in spectrum of tribenzocyclyne; *cf.* band from AEM-**1** at 1598.7.
1583.4 (w)	C=C stretch in typical region for fused aromatics. Also present in spectrum of tribenzocyclyne; *cf.* band from AEM-**1** at 1579.8.

1513.4 (m)	Phenyl ring C=C vibrational mode. Characteristic band. Shifted by 1.0 cm^{-1} from benzene 1,4-diboronic acid.
1483.1 (m)	C=C vibrational modes for triphenylene building block. These are characteristic bands for triphenylene. Shifted by − 26.1 cm^{-1}.
1384.7 (m)	B−O stretch, characteristic band for boroxoles.
1334.9 (s)	B−O stretch, characteristic band for boroxoles.
1238.9 (m)	C−O stretch, characteristic for boroxoles.
1186.7 (m)	C−H in plane bending modes.
1078.1 (m)	
1019.0 (m)	B−C stretch.
869.3 (m)	C−H out of plane bands for *p*-substituted aromatic
833.6 (m)	
663.2 (m)	

7. FT-IR spectra of AEM-COF-2, AEM-2 and BDBA

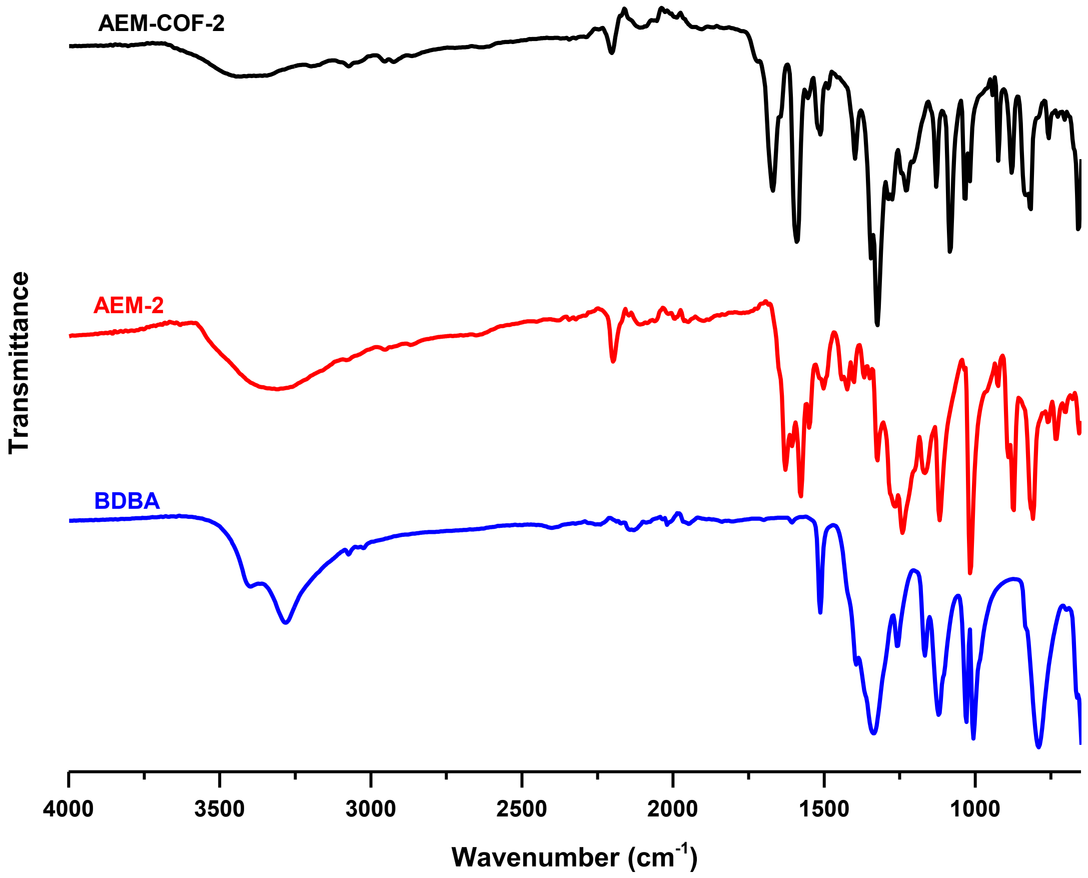

Figure S4. FT-IR spectra of "as synthesized" AEM-COF-**2**, AEM-**2,** and BDBA.

8. Peak assignments for FT-IR spectrum of AEM-COF-2

Table S2. Peak assignments for FT-IR spectrum of AEM-COF-**2**

Peak (cm^{-1})	Assignment and Notes
3432.1 (m)	O-H stretch from or end –$B(OH)_2$ groups at the surface of crystallites.
3073.8 (w)	Aromatic C−H stretch from benzene 1,4-diboronic acid phenyl group of AEM-COF-**2**; *cf.* band from benzene 1,4-diboronic acid at 3074.3 (w).
2954.7 (w)	C−H stretch from phenanthrene group of AEM-**2** of AEM-COF-**2**.
2925.7 (w)	
2863.7 (w)	
2202.1 (w)	C≡C stretch from AEM-**2** of AEM-COF-**2**; *cf.* band from AEM-**2** monomer at 2198.4.
1669.8 (m)	C=C stretch from AEM-**2** of AEM-COF-**2**; *cf.* band from AEM-**2** at 1628.2.
1589.5 (s)	C=C stretch from AEM-**2** of AEM-COF-**2**; *cf.* band from AEM-**2** at 1577.2.
1512.9 (m)	C=C vibrational mode of benzene 1,4-diboronic acid phenyl groups of AEM-COF-**2**. Could also be overlapped with some bands from the AEM-**2** building blocks of AEM-COF-**2**.
1397.5 (m)	B−O stretch, characteristic band for boroxoles.
1345.1 (s)	B−O stretch, characteristic band for boroxoles. Could also be overlapped with some bands from the AEM-**2** building blocks of AEM-COF-**2**.
1323.0 (s)	C−O stretch, characteristic for boroxoles. Could also be overlapped with some bands from the AEM-**2** building blocks of AEM-COF-**2**.
1228.2 (m)	C−O stretch, characteristic for boroxoles.
1029.0 (m)	C−H in plane bending modes.
1084.1 (m)	
1033.9 (m)	B−C stretch.
1017.4 (m)	
924.6 (m)	C−H out of plane bands
879.0 (m)	
817.2 (m)	

9. Powder X-Ray Diffraction Patterns of AEM-COF-1, AEM-1, and BDBA

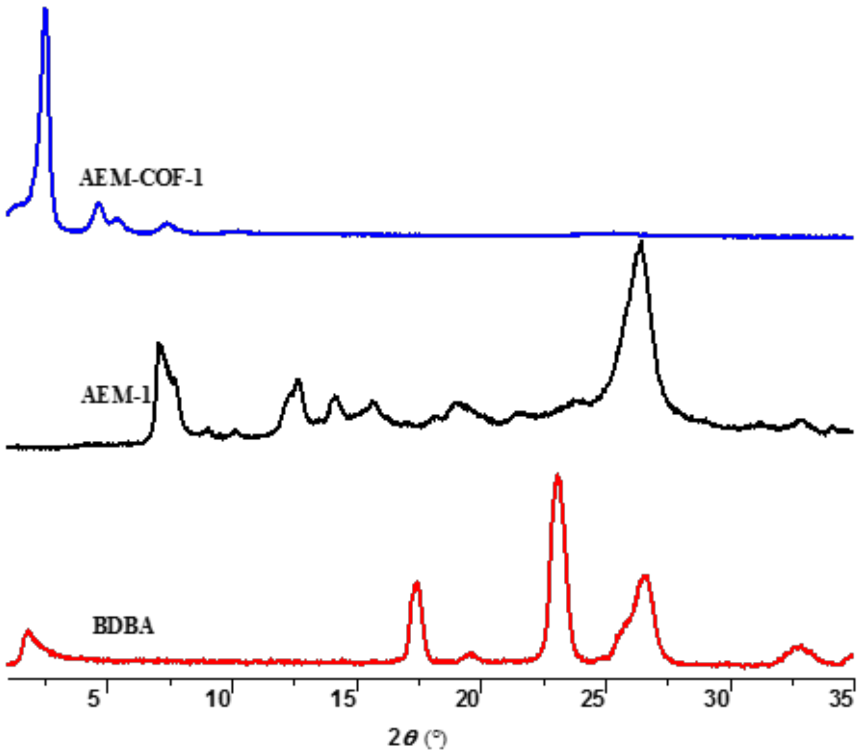

Figure S5. PXRD patterns of AEM-COF-**1**, AEM-**1**, and BDBA.

10. Powder X-Ray Diffraction Patterns of AEM-COF-2, AEM-2, and BDBA

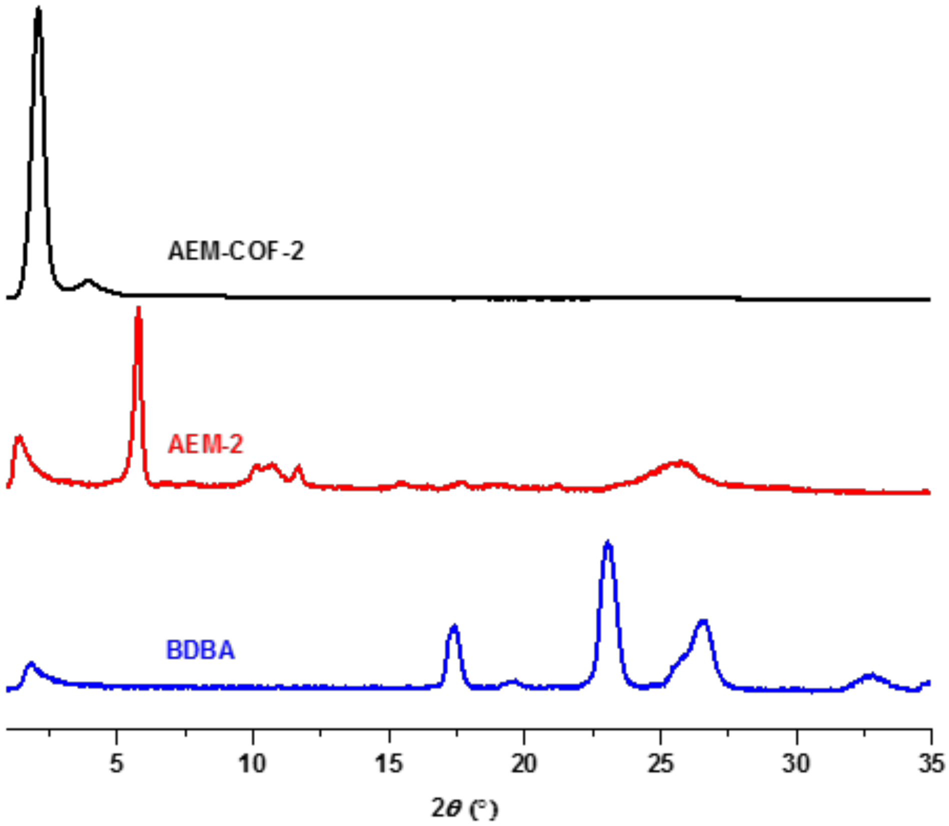

Figure S6. PXRD patterns of AEM-COF-**2**, AEM-**2**, and BDBA.

11. Structural Modeling and X-ray Diffraction Analyses.

All the models, including cell parameters and atomic positions were generated using the *Materials Studio* software package, employing the *Materials Visualizer* module. All the models were constructed in the hexagonal system, with the layers lying on the **ab** plane. The space groups with the maximum possible symmetry were selected. An energy minimization was performed to optimize the geometry of the building units, employing the universal force field implemented in the *Forcite* module of *Materials Studio*. During this process, the unit cell parameters for each model were also optimized. In Table S1, the values of the optimized unit cell parameters and the space group for the models constructed are summarized.

Table S3. Optimized unit cell parameters of the constructed models for AEM-COF-**1** and AEM-COF-**2**.

	AEM-COF-**1**		AEM-COF-**2**	
	eclipsed	staggered	eclipsed	staggered
**Space group**	*P6/mmm*	$P6_3/mmc$	*P6/mmm*	$P6_3/mmc$
***a* (Å)**	35.528	35.519	40.935	39.598
***c* (Å)**	3.398	6.477	3.257	6.420

The corresponding powder patterns for the four models were calculated and compared with the experimental patterns, finding the best agreement for the eclipsed models. With them, full profile pattern (Pawley) refinements were performed against the experimental powder patterns obtaining the refined unit cell parameters. Tables S2 and S3 shows the refined cell parameters and the fractional atomic coordinates of the two final models.

Table S4. Refined unit cell parameters and fractional atomic coordinates for AEM-COF-**1**.

**Name**	AEM-COF-**1**		
**Space group**	*P6/mmm*		
***a* (Å)**	35.528		
***c* (Å)**	3.398		
**Atom name**	***x***	***y***	***z***
H1	0.03993	0.58096	1.00000
C2	0.02280	0.54563	1.00000
C3	0.04583	0.52291	1.00000
B4	0.09679	0.54839	1.00000
O5	0.12285	0.59611	1.00000
C6	0.16443	0.60164	1.00000
C7	0.20323	0.64152	1.00000
H8	0.20249	0.67173	1.00000
C9	0.24298	0.64154	1.00000
C10	0.28330	0.68267	1.00000

Table S5. Refined unit cell parameters and fractional atomic coordinates for AEM-COF-**2**.

**Name**	AEM-COF-**2**		
**Space group**	*P6/mmm*		
***a* (Å)**	40.935		
***c* (Å)**	3.257		
**Atom name**	***x***	***y***	***z***
H1	0.03229	0.56547	1.00000

C2	0.01843	0.53690	1.00000
C3	0.03705	0.51853	1.00000
B4	0.07824	0.53912	1.00000
O5	0.09932	0.57761	1.00000
C6	0.13305	0.58220	1.00000
C7	0.16440	0.61456	1.00000
C8	0.16354	0.64617	1.00000
H9	0.13867	0.64591	1.00000
C10	0.19481	0.67849	1.00000
H11	0.19374	0.70267	1.00000
C12	0.22721	0.67959	1.00000
C13	0.25952	0.71296	1.00000

12. Solid State NMR spectra of the as synthesized AEM-COF-1 and AEM-COF-2

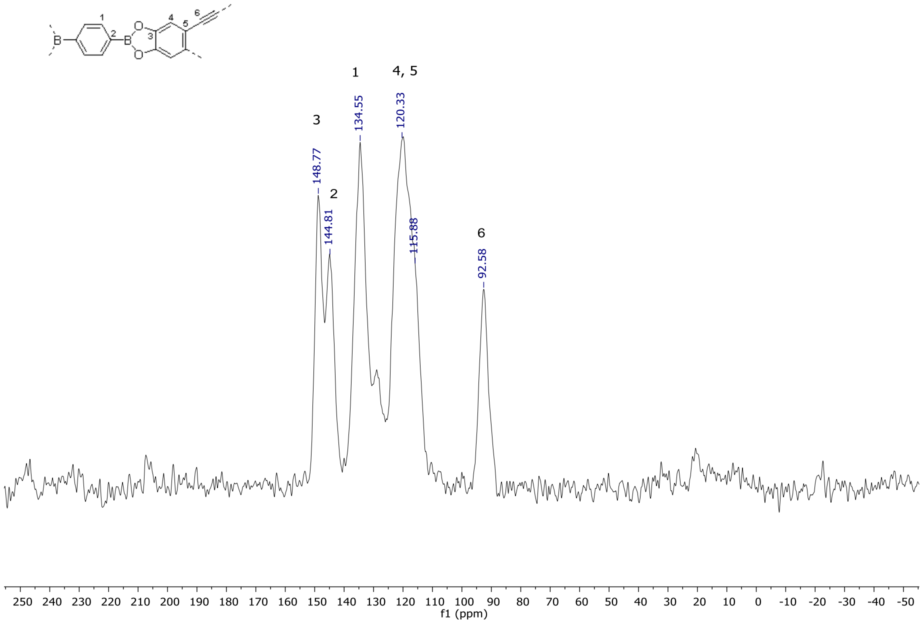

Figure S7. ^{13}C-CPMAS spectrum of the AEM-COF-**1**.

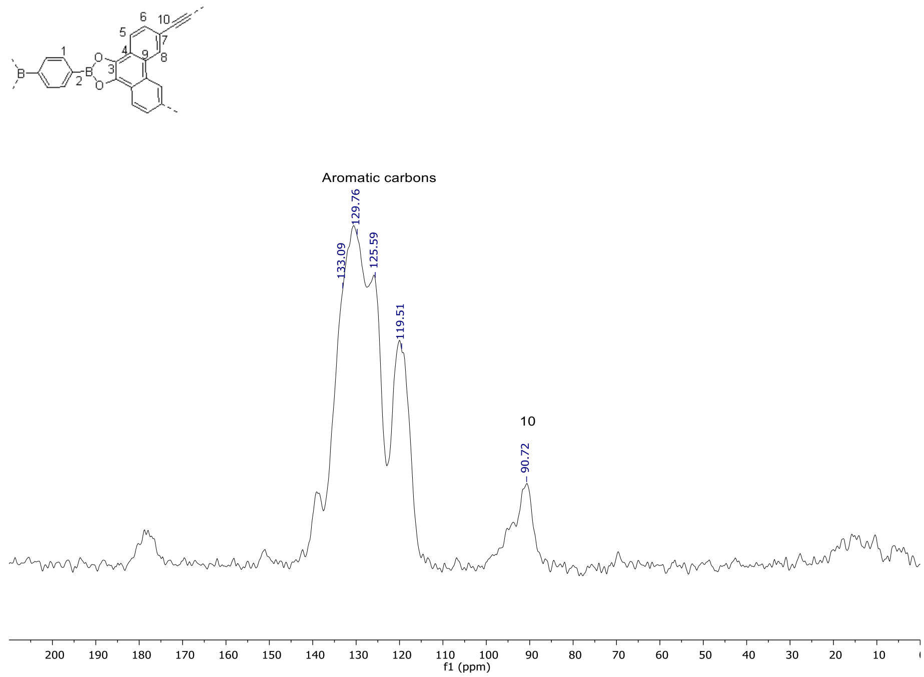

Figure S8. ^{13}C-CPMAS spectrum of AEM-COF-**2**.

13. ^{1}H and ^{13}C NMR spectra of the new compounds

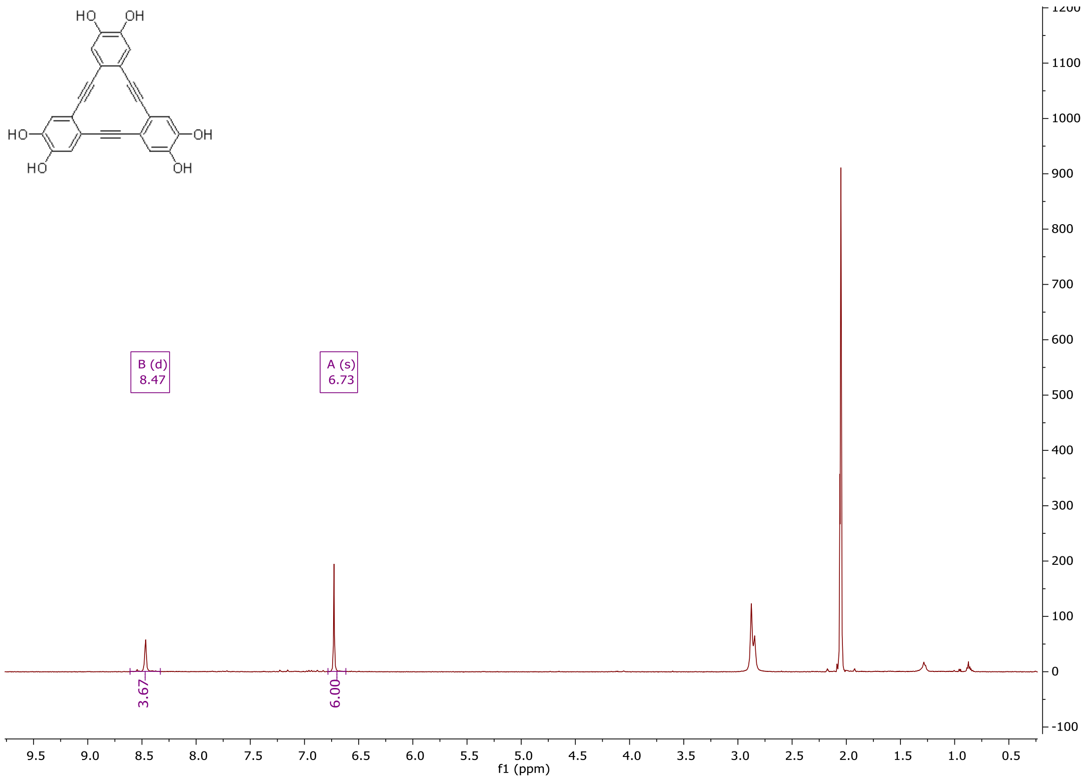

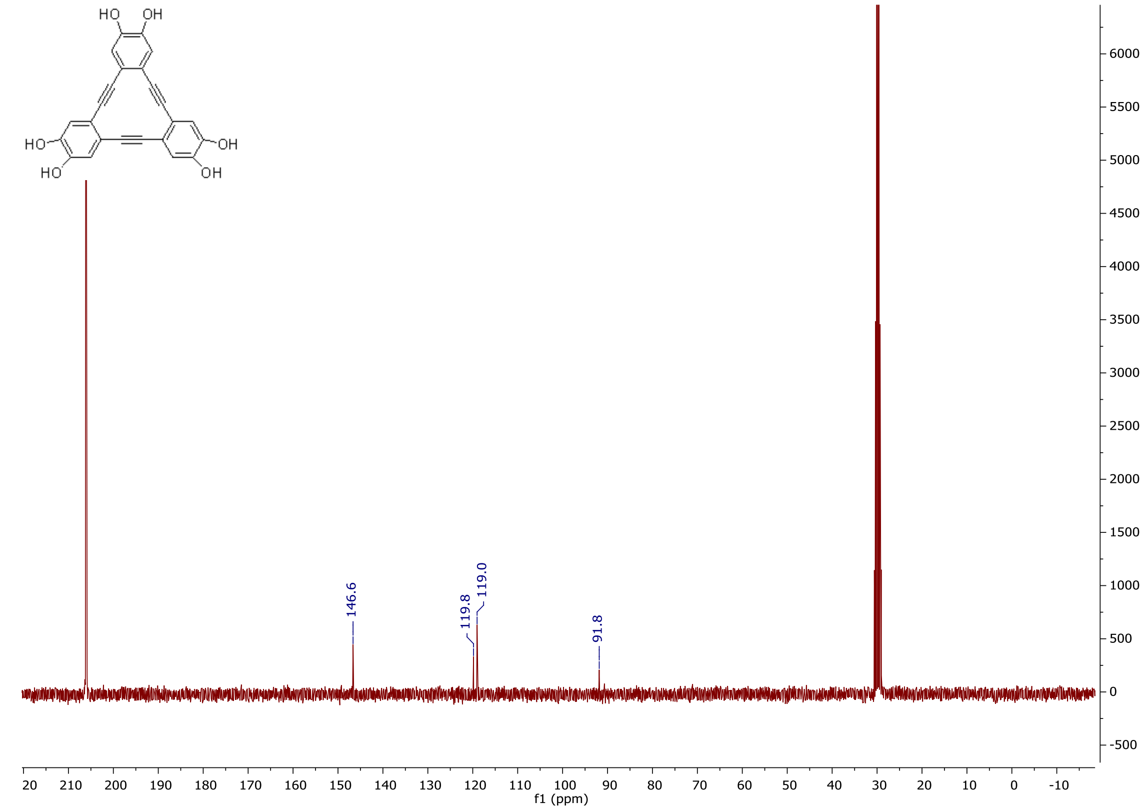
HO
OH
HO
OH
HO
OH
146.6
119.8
119.0
91.8
6000
5500
5000
4500
4000
3500
3000
2500
2000
1500
1000
500
0
-500
20
210
200
190
180
170
160
150
140
130
120
110
100
90
80
70
60
50
40
30
20
10
0
-10
f1 (ppm)

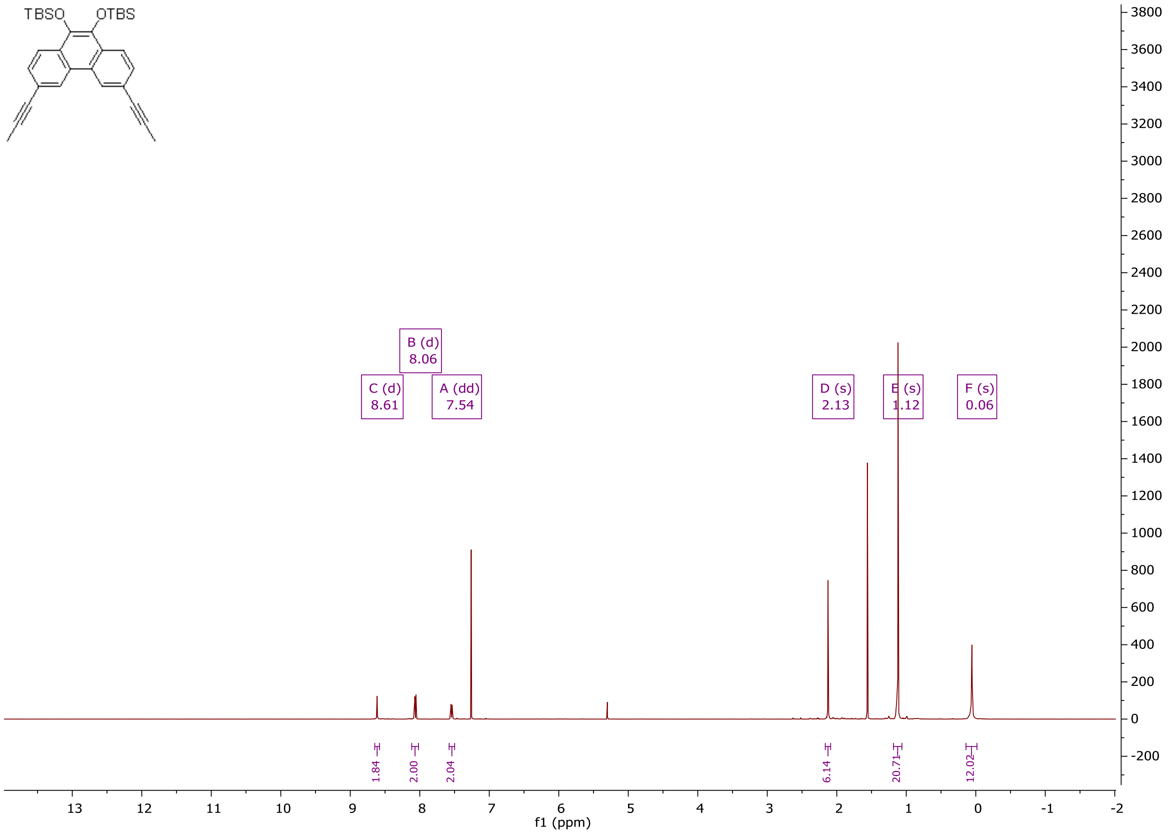
TBSO
OTBS
B (d)
8.06
C (d)
8.61
A (dd)
7.54
D (s)
2.13
E (s)
1.12
F (s)
0.06
1.84
2.00
2.04
6.14
20.71
12.02
3800
3600
3400
3200
3000
2800
2600
2400
2200
2000
1800
1600
1400
1200
1000
800
600
400
200
0
-200
13
12
11
10
9
8
7
6
5
4
3
2
1
0
-1
-2
f1 (ppm)

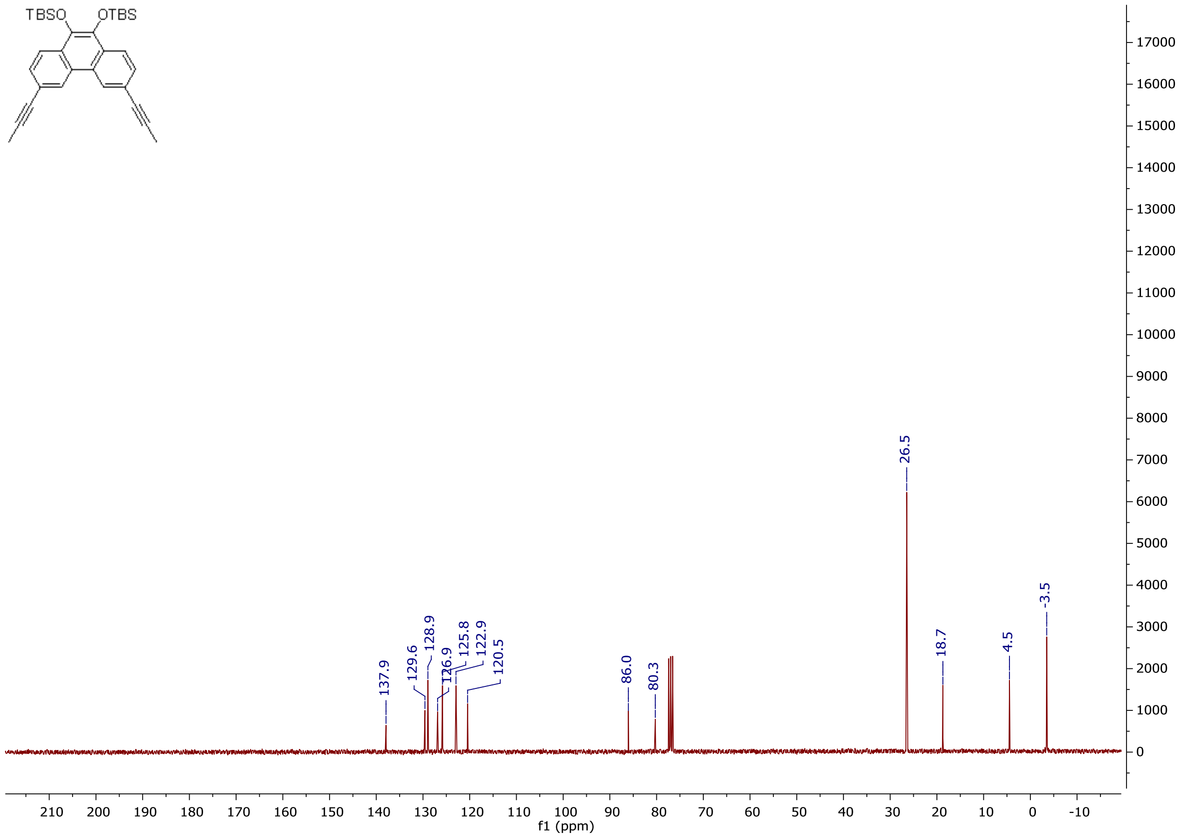
TBSO
OTBS
137.9
129.6
128.9
126.9
125.8
122.9
120.5
86.0
80.3
26.5
18.7
4.5
-3.5
f1 (ppm)

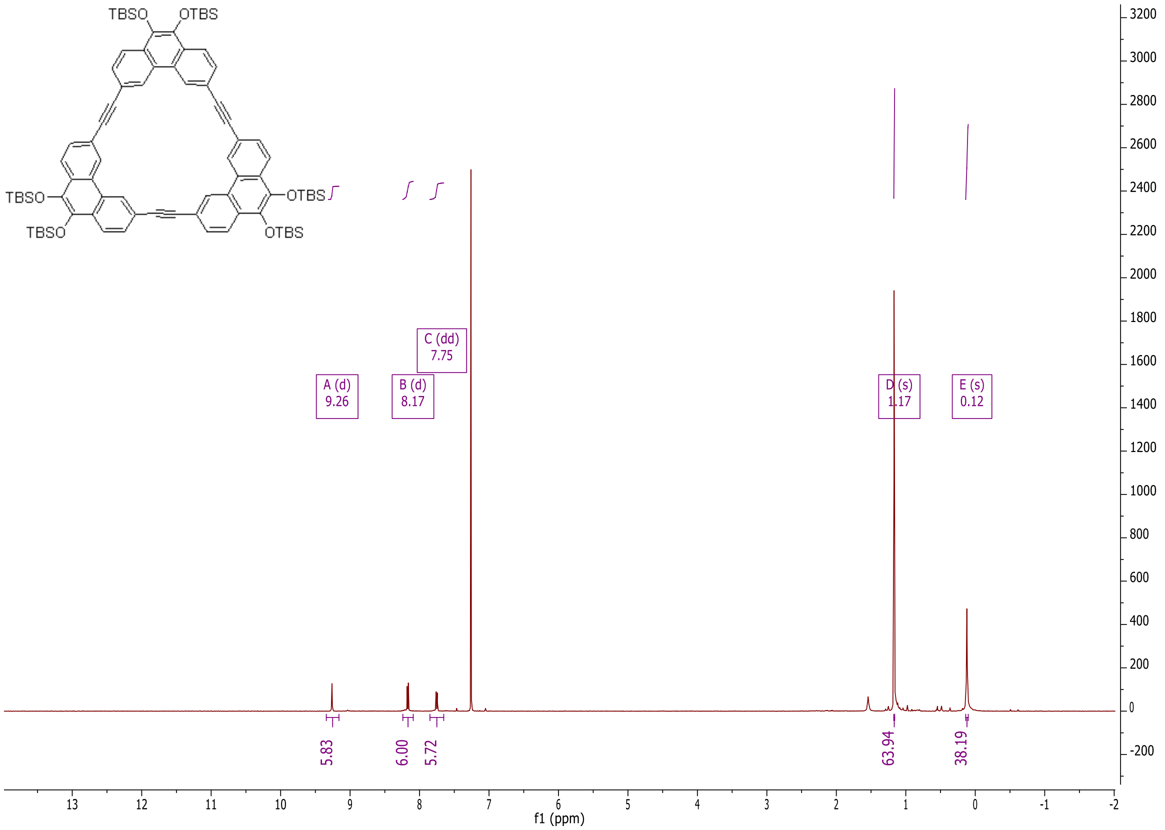
TBSO
OTBS
A (d)
9.26
B (d)
8.17
C (dd)
7.75
D (s)
1.17
E (s)
0.12
5.83
6.00
5.72
63.94
38.19
f1 (ppm)

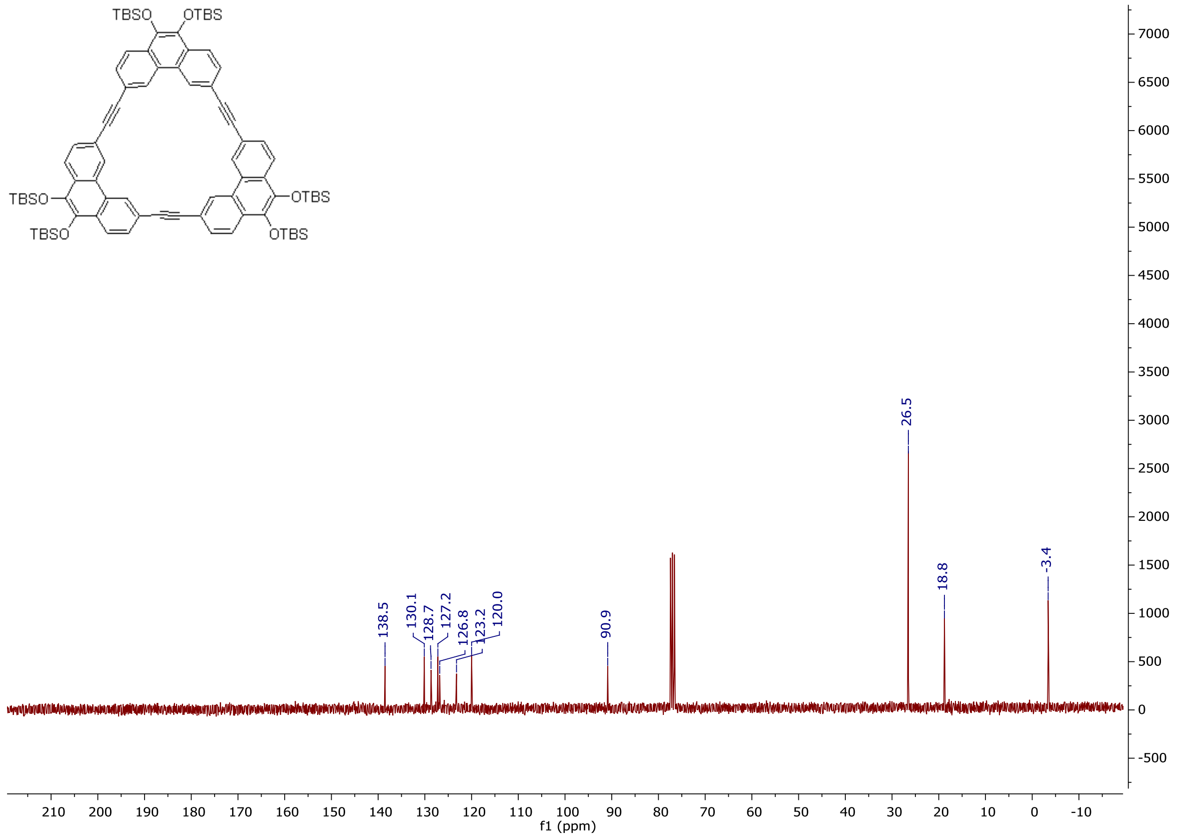
TBSO
OTBS
TBSO
OTBS
TBSO
OTBS
138.5
130.1
128.7
127.2
126.8
123.2
120.0
90.9
26.5
18.8
-3.4
f1 (ppm)

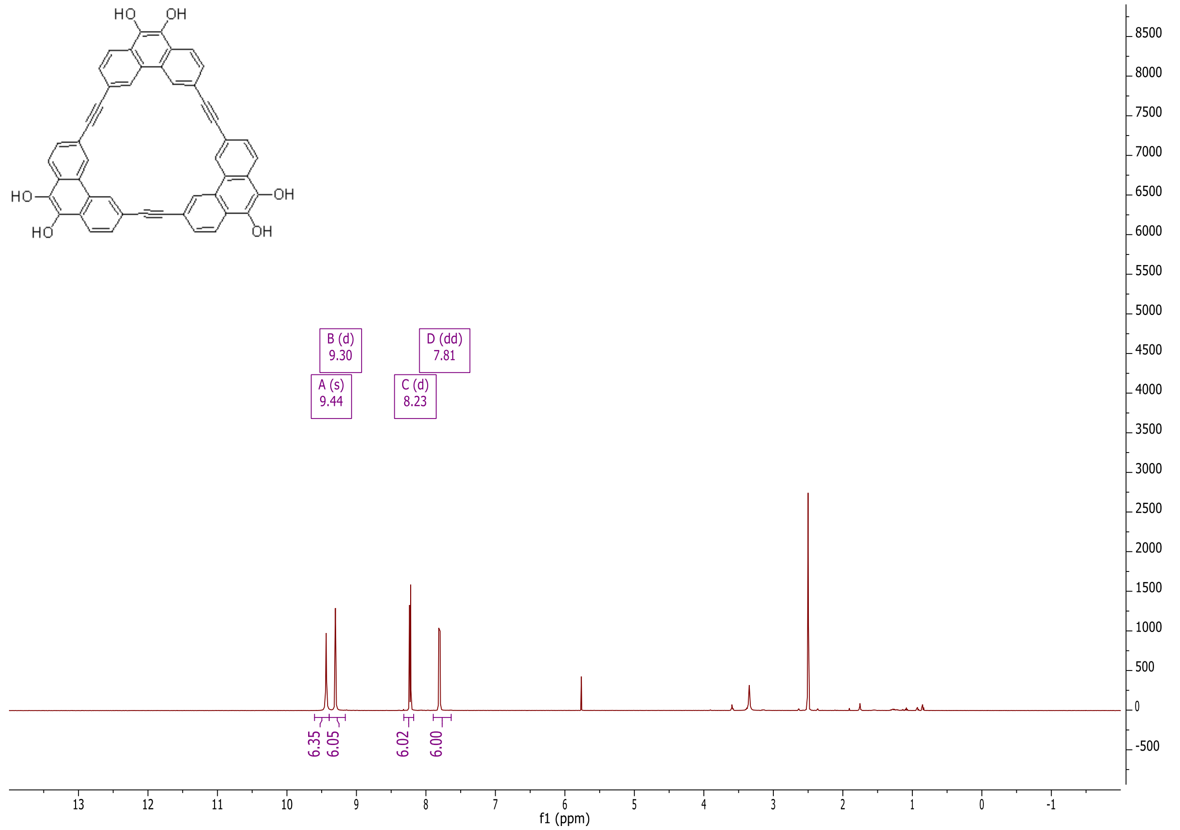
HO
OH
HO
OH
HO
OH
B (d)
9.30
A (s)
9.44
C (d)
8.23
D (dd)
7.81
6.35
6.05
6.02
6.00
f1 (ppm)

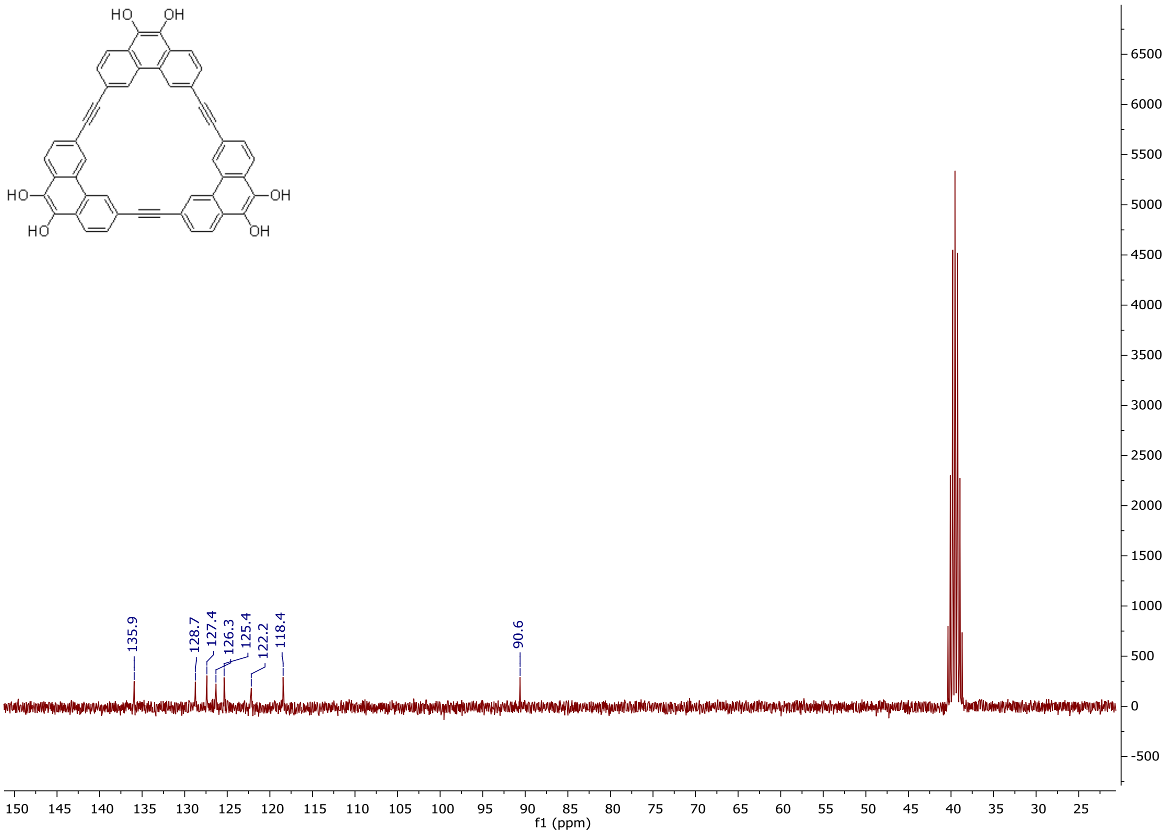

(1) H. Yang, Z. Liu, and W. Zhang, *Adv. Synth. Catal.* 2013, **355**, 885.
(2) B. Kobin, L. Grubert, S. Blumstengel, F. Henneberger, and S. Hecht, *J. Mater. Chem.* 2012, **22**, 4383.
(3) J. M. Fox, N. R. Goldberg, and T. J. Katz, *J. Org. Chem.* 1998, **63**, 7456.
